# Surgical hip dislocation with relative femoral neck lengthening and retinacular soft-tissue flap for sequela of Legg–Calve–Perthes disease

**DOI:** 10.1007/s00064-022-00780-9

**Published:** 2022-08-05

**Authors:** Christiane Sylvia Leibold, Nicolas Vuillemin, Lorenz Büchler, Klaus Arno Siebenrock, Simon Damian Steppacher

**Affiliations:** Department of Orthopaedic Surgery and Traumatology, Inselspital, Bern University Hospital, University of Bern, Freiburgstr. 18, 3010 Bern, Switzerland

**Keywords:** Perthes disease, Surgical hip dislocation, Relative neck lengthening, High riding trochanter, Trochanteric distalization, Morbus Perthes, Chirurgische Hüftluxation, Relative Schenkelhalsverlängerung, Trochanterdistalisierung, Trochanterhochstand

## Abstract

**Objective:**

Correction of post-LCP (Legg–Calve–Perthes) morphology using surgical hip dislocation with retinacular flap and relative femoral neck lengthening for impingent correction reduces the risk of early arthritis and improves the survival of the native hip joint.

**Indications:**

Typical post-LCP deformity with external and internal hip impingement due to aspherical enlarged femoral head and shortened femoral neck with high riding trochanter major without advanced osteoarthritis (Tönnis classification ≤ 1) in the younger patient (age < 50 years).

**Contraindications:**

Advanced global osteoarthritis (Tönnis classification ≥ 2).

**Surgical technique:**

By performing surgical hip dislocation, full access to the hip joint is gained which allows intra-articular corrections like cartilage and labral repair. Relative femoral neck lengthening involves osteotomy and distalization of the greater trochanter with reduction of the base of the femoral neck, while maintaining vascular perfusion of the femoral head by creation of a retinacular soft-tissue flap.

**Postoperative management:**

Immediate postoperative mobilization on a passive motion device to prevent capsular adhesions. Patients mobilized with partial weight bearing of 15 kg with the use of crutches for at least 8 weeks.

**Results:**

In all, 81 hips with symptomatic deformity of the femoral head after healed LCP disease were treated with surgical hip dislocation and offset correction between 1997 and 2020. The mean age at operation was 23 years; mean follow-up was 9 years; 11 hips were converted to total hip arthroplasty and 1 patient died 1 year after the operation. The other 67 hips showed no or minor progression of arthrosis. Complications were 2 subluxations due to instability and 1 pseudarthrosis of the lesser trochanter; no hip developed avascular necrosis.

## Abstract (de)

**Operationsziel:**

Korrektur der Pathomorphologie des proximalen Femurs nach durchgemachtem Morbus Perthes mittels chirurgische Hüftluxation mit retinakulärem Weichteillappen und relativer Schenkelhalsverlängerung, um ein Impingement zu korrigieren, die Ausbildung einer frühen Arthrose zu reduzieren und die Überlebenszeit des nativen Hüftgelenks zu verlängern.

**Indikationen:**

Typische Deformität nach Morbus Perthes mit extra- und intraartikulärem Impingement durch asphärischen, vergrößerten Kopf und verkürzten Schenkelhals mit relativem Trochanterhochstand ohne fortgeschrittene Degeneration (Tönnis-Klassifikation ≤ 1) bei jüngeren Patienten (Alter < 50 Jahre).

**Kontraindikationen:**

Fortgeschrittene globale Gelenkdegeneration (Tönnis-Klassifikation ≥ 2).

**Operationstechnik:**

Mittels chirurgischer Hüftluxation kann das komplette Hüftgelenk eingesehen werden, und intraartikuläre Korrekturen wie Knorpel- und Labrumreparaturen können durchgeführt werden. Die relative Schenkelhalsverlängerung beinhaltet die Osteotomie und Distalisierung des Trochanter major in Kombination mit Reduktion der Basis des Schenkelhalses unter Schonung der Gefäßversorgung des Femurkopfs durch Entwicklung eines retinakulären Weichteillappens.

**Weiterbehandlung:**

Direkt postoperativ Mobilisation auf einer passiven Bewegungsschiene zur Vorbeugung von kapsulären Adhäsionen. Für mindestens 8 Wochen findet nur eine Teilbelastung des Beins statt.

**Ergebnisse:**

Es wurden 81 Hüften mit symptomatischer Deformität des Femurkopfs nach Morbus Perthes mittels chirurgischer Hüftluxation im Zeitraum von 1997 bis 2020 behandelt. Das mittlere Alter bei Operation betrug 23 Jahre, der mittlere Nachkontrollzeitraum für alle Patienten war 9 Jahre. Bei 11 Hüften erfolgte die Implantation einer Hüfttotalprothese, 1 Patient war 1 Jahr nach der Operation verstorben. Die weiteren 67 Hüften zeigten keine oder eine geringe Progression der Arthrose. Komplikationen traten auf bei 2 Hüften die eine postoperative Subluxation zeigten und 1 Pseudarthrose des Trochanter minor; eine avaskuläre Nekrose zeigte sich bei keiner Hüfte.

## Introductory remarks

Legg–Calve–Perthes (LCP) disease is an aseptic osteonecrosis of the femoral head of the developing hip. The course of the disease can be divided into typical stages according to Waldenström (Infobox; [9, 20]). The disease shows an inhomogeneous appearance. Some patients show only mild deformations and have little or no late effects, some are affected more severely with early development of arthritis, and some patients are limited in their daily life even before developing osteoarthritis due to impaired range of motion of the hip joint. Around 20% of patients are affected bilaterally [12]. The altered shape of the proximal femur after LCP disease can cause restricted range of hip motion, femoroacetabular impingement, hip pain, and joint degeneration in young adults [3, 5]. The typical post-LCP deformity in the mature hip consists of a mushroom-shaped head, femoral neck shortening and relatively high riding trochanter. Additional acetabular deformities such as hip dysplasia or retroversion are frequent. Functional problems can result due to femoral or acetabular pathomorphology.

Femoral functional problems can result from the intraarticular cam impingement due to the aspherical enlarged femoral head. If the head is too large to slip under the acetabular rim a head-induced pincer impingement with hinged abduction will occur. In addition, functional retrotorsion of the femur with intraarticular impingement can also occur.

Other femoral functional problems are the extraarticular impingement of the greater trochanter with limited external rotation in flexion and abduction or the extraarticular impingement of the lesser trochanter on the ischial tubercle with limited external rotation in extension. Typical acetabular pathologies consist of dysplasia, acetabular retroversion, and incongruity [17]. The choice of which surgical treatments to use depends on the pathomorphology of the individual patient and requires a stepwise treatment pathway [7].

In this article, we will focus on surgical hip dislocation with relative femoral neck lengthening for the correction of the intra- and extraarticular impingement and further the development of a retinacular soft-tissue flap for the maintenance of femoral head perfusion [2, 4, 8, 11].

## Surgical principle and objective

**Fig. 1:**
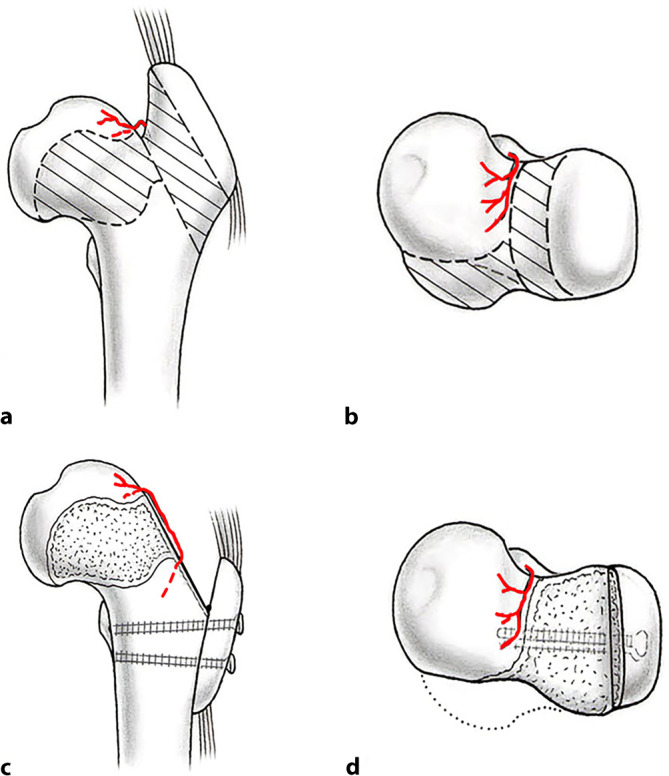
Post-Legg–Calve–Perthes (LCP) deformity of the hip. **a** The typical post-LCP deformity of the hip includes an aspherical and enlarged head with a shortened femoral neck. This results in a relatively high riding greater and lesser trochanter potentially resulting in extraarticular impingement. **b** In addition, the aspherical head results in intraarticular impingement. The area of bone resection (*dashed area*) is close to the nutrient vessel of the femoral head (medial femoral circumflex artery in* red*). **c** To elongate the femoral neck with the goal to overcome extraarticular impingement, a retinacular flap is developed to maintain femoral head perfusion. **d** The greater trochanter is refixated in a distalized position and the femoral neck is reshaped with offset correction in close proximity to the nutrient vessel. (From [17], with kind permission from SAGE Publishing)

The aim of surgical hip dislocation with relative femoral neck lengthening is to correct intra- and extraarticular femoroacetabular impingement. Surgical hip dislocation allows an excellent overview of the complete hip joint and allows for intraoperative dynamic testing to evaluate impingement-free range of motion of the hip. In addition, intraarticular corrections like labral and cartilage repair are possible. The relative femoral neck lengthening allows distalization of the greater trochanter and reduction of its base to elongate the femoral neck (Fig. 1). This improves range of motion and corrects intra- and extraarticular impingement of the femoral neck or greater trochanter, respectively. Therefore, a retinacular soft-tissue flap has to be developed to secure femoral head perfusion. For this procedure, precise knowledge of the vascular anatomy of the proximal femur is essential to avoid iatrogenic necrosis [10, 14]. Other deformities such as the head-induced pincer impingement with hinged abduction can be corrected with an additional head reduction osteotomy [2, 16]. The extraarticular impingement of the lesser trochanter can be corrected by distalization.

## Advantages

- Surgical hip dislocation [6] provides unrestricted access to the acetabulum and the femoral head including dynamic intraoperative testing of range of motion and impingement.
- With development of the retinacular flap, the femoral head perfusion is secured and deformities of the head–neck region close to the retinacular vessels can be corrected [8, 11, 19].
- The relative femoral neck lengthening allows distalization of the greater trochanter and reduction of its base to elongate the femoral neck. The goal is to restore range of motion without extra- or intraarticular impingement.
- Concomitant procedures such as transposition of the lesser trochanter, head reduction osteotomies, treatment of cartilage and labral lesions or femoral torsion can be performed [18].

## Disadvantages

- Technically demanding surgical technique. Without precise knowledge of the vascular anatomy of the proximal femur, there is the risk of iatrogenic necrosis of the femoral head.
- Rehabilitation with a minimum 8 weeks of limited weight bearing.
- Changes in the abductor lever arm may lead to long rehabilitation with limping.
- Trochanteric screws are often irritating requiring hardware removal.
- No true leg lengthening

## Indications

- Post-LCPD(Legg-Calve-Perthes Disease) deformity of the hip with a short femoral neck, aspherical femoral head, and a relative high riding trochanter resulting in extra- and intraarticular hip impingement.
- No advanced degenerative signs (Tönnis grade ≤ 1)
- Relatively young patient (age < 50 years)

## Contraindications

- Advanced joint degeneration (Tönnis grade ≥ 2)
- Elderly patients (age ≥ 50 years)

## Patient information

- General surgical risks:
  (Thrombosis, pulmonary embolism, allergic reactions, injury of cutaneous nerves with numbness/dysesthesia, excessive bleeding with need of blood transfusion, delayed wound healing and infection)
- Specific risks of this procedure:
  - Delayed union or pseudarthrosis of osteotomy of greater trochanter
  - Intraarticular adhesions
  - Heterotopic ossifications
  - Iatrogenic avascular necrosis due to damage of the retinacular vessels

## Preoperative work-up

- Detailed clinical and radiological work-up is essential to evaluate whether hip-preserving surgery is indicated. As an alternative, total hip arthroplasty (THA) has to be considered in hips with advanced osteoarthritis.
- Detailed patient history (e.g., family history, onset and course of disease, symptoms, previous treatment and operations).
- Standardized radiographic imaging including an anteroposterior pelvic radiograph and axial view.
- Magnetic resonance (MR) arthrography of the hip, preferably with radial reconstruction and intraarticular contrast injection for evaluation of size and location of cam deformity, congruency of the joint, deformity of greater and lesser trochanter with extraarticular impingement and damage to cartilage or labrum [13].
- Axial imaging (computed tomography or magnetic resonance imaging) of hip and knees for evaluation of femoral torsion.
- Abduction/internal rotation radiograph for evaluation of congruency or instability of the joint or hinge abduction.
- Preoperative templating to define the location of the osteotomy, the type of correction (relative femoral neck lengthening and offset correction or additional femoral head reduction, femoral osteotomy or acetabular osteotomy).

## Instruments

- Dedicated instruments for hip preservation surgery with special retractors (Subtilis; Accuratus, Bern, Switzerland)
- Cortical 3.5 mm screws for refixation of the greater trochanter
- Sterile bag on contralateral side to position the leg during surgical hip dislocation
- Intraoperative fluoroscopy (optional)
- Anchor sutures for labral reattachment (Gryphon Suture Anchors, DePuy Synthes, Zuchwil, Switzerland)
- Fibrin glue (Tissuecol; Baxter; Warsaw, Poland) or AMIC (Autologous Matrix-Induced Chondrogenesis) with type I/III collagen matrix (Chondro-Gide; Geistlich Pharma, Wollhusen, Switzerland) for cartilage treatment

## Anesthesia and positioning

- General anesthesia with full muscle-relaxation
- Lateral decubitus position with placement of the leg on a tunnel bolster to avoid pressure on contralateral leg and to have a flat surface in a horizontal position for the involved lower limb
- Stabilization of patient with two side supports
- Disinfection and sterile drapes including the entire lower extremity up to the thorax. The greater trochanter should be freely palpable.
- Fluoroscopy for intraoperative orientation and monitoring of the osteotomy, angular corrections and placement of hardware
- Single-shot intravenous antibiotic prophylaxis (cefazolin 2 g i.v.)

## Surgical technique

### Surgical hip dislocation

**Fig. 2:**
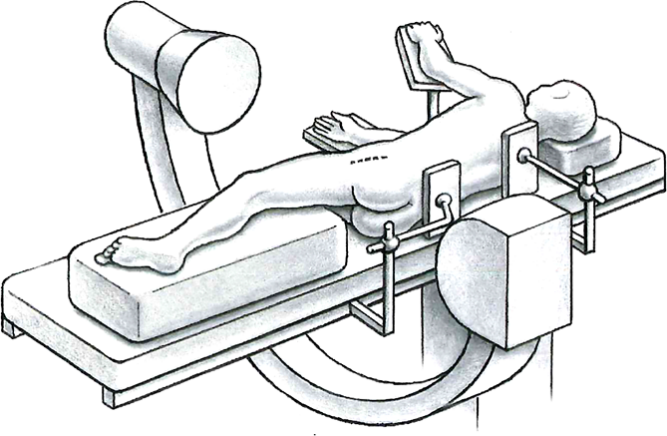
A straight lateral skin incision of 15–20 cm is made, centered over the greater trochanter with the patient in the lateral decubitus position. (With kind permission from Dr. Hermann Oberli)

**Fig. 3:**
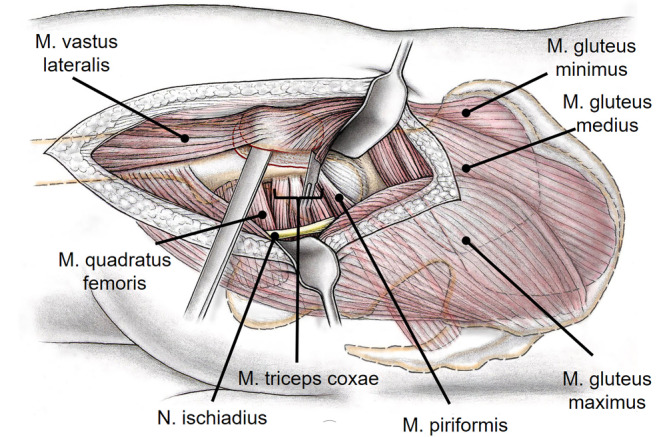
After incision of the iliotibial band, the superficial interval between gluteus maximus and medius (Gibson interval) is developed. The deep interval lies between the piriformis and the gluteus minimus muscle and is best developed with the hip in extension and internal rotation. A flat trochanteric osteotomy is performed to facilitate the distalization of the greater trochanter (for surgical hip dislocations without distalization of the greater trochanter usually a stepped osteotomy is performed to minimize the risk of trochanteric pseudarthrosis after refixation). (From [22], with kind permission from Oxford University Press)

**Fig. 4:**
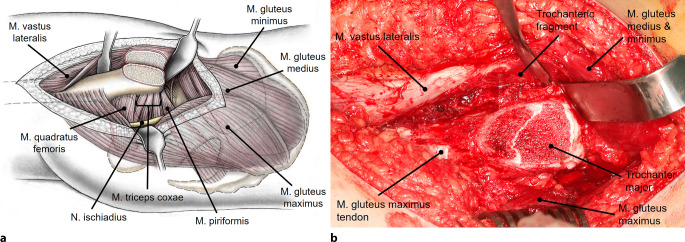
The osteotomy starts at the posterosuperior tip of the greater trochanter and ends distally 10–15 mm to the lateral tubercle. Proximally, the osteotomy should end just behind the most posterior insertion of the gluteus medius leaving the short external rotators attached to the proximal femur. To avoid injury to the retinatcular vessels, care has to be taken not to penetrate the fossa piriformis. The trochanteric fragment is mobilized anterior together with the origin of the vastus lateralis and the insertion of the gluteus minimus and medius muscle. **a** Schematic drawing after removal of the trochanteric fragment. **b** Intraoperative view after removal of the trochanteric fragment. The capsule is exposed by mobilizing the gluteus minimus from the capsule. (**a** from [22], with kind permission from Oxford University Press)

**Fig. 5:**
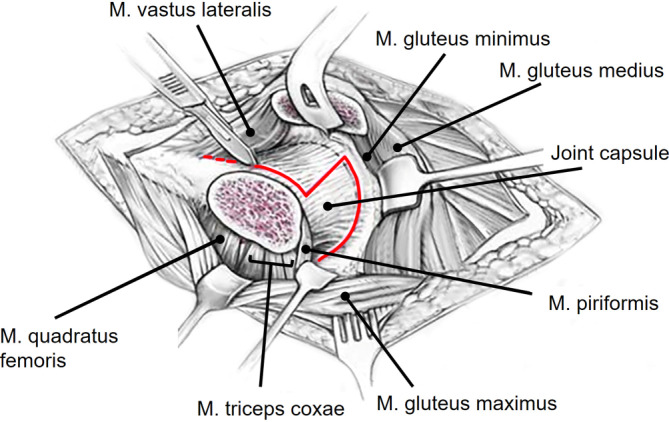
The capsule is incised in a z-shaped manner (*red line*) without injuring the labrum or the retinacular vessels at the posterosuperior aspect of the femoral neck. (From [23], with kind regards from Springer Nature)

**Fig. 6:**
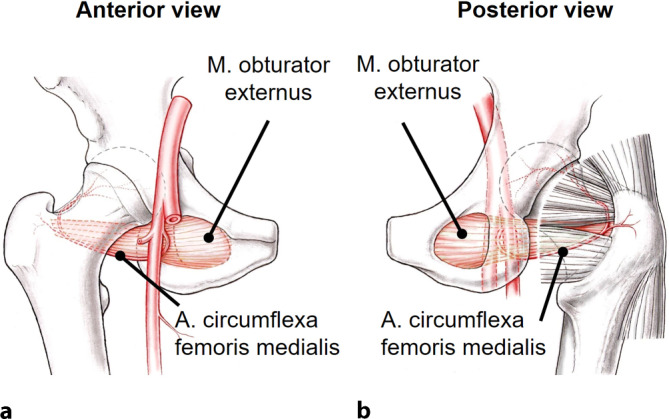
The vascular anatomy of the proximal femur has to be preserved when developing the retinacular flap for the relative femoral neck lengthening. **a** The main nutrient vessel of the femoral head is the medial branch of the medial femoral circumflex artery (MCFA). It originates from the deep femoral artery, runs along the inferior border of the obturator externus muscle towards the intertrochanteric crest. **b** Posterior view of the hip: Crossing the obturator externus tendon dorsal and the triceps coxae muscle anterior, the terminal branches of the MCFA run along the posterosuperior aspect of the femoral neck and perforate the head as the retinacular vessels at the cartilage border. (From [24], with kind regards from Springer Nature)

Figs. 2, 3, 4, 5, and 6.

### Development of retinacular flap and relative femoral neck lengthening

**Fig. 7:**
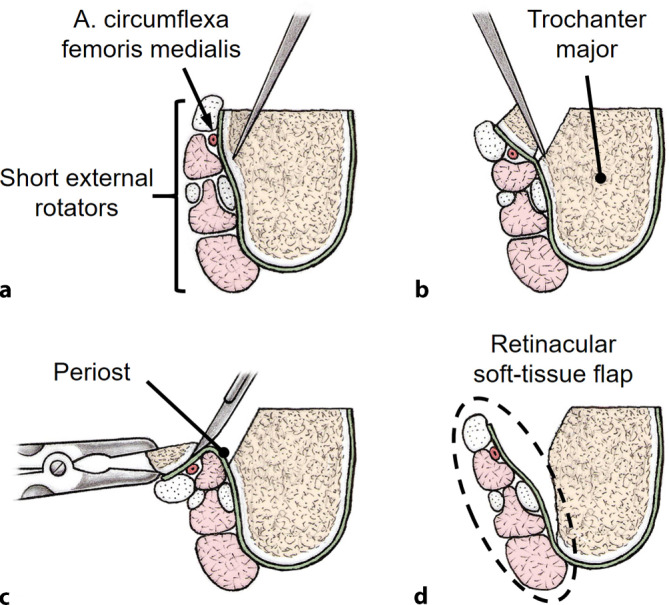
Cross-section view of the femoral neck: The soft-tissue flap contains the nutrient vessels to the femoral head and is developed in a stepwise, subperiosteal resection of the greater trochanter and the posterosuperior neck. For the relative femoral neck lengthening, the anterior portion of the femoral neck does not need to be prepared as opposed to the description of the retinacular soft-tissue flap for slipped capital epiphysiolysis [21]. (**a** from [25], with kind permission of Universimed Cross Media Content GmbH, **b** From [22], with kind permission from Oxford University Press)

**Fig. 8:**
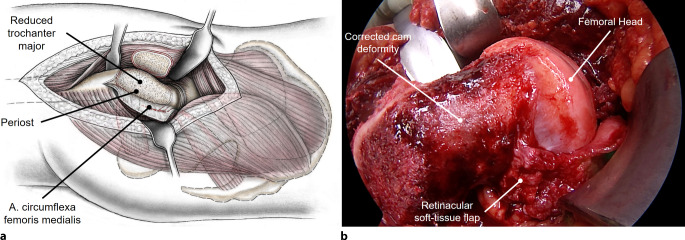
The ligament of the femoral head is cut after traction of the femur to allow dislocation of the femoral head and inspection of the joint. The leg is positioned in flexion and external rotation in a plastic bag on the contralateral side of the operation table to gain maximal access to the femur. Now the femoral head–neck junction can be approached. Trimming of the head neck junction to correct the cam deformity with chisels and a high-speed burr to correct the intraarticular impingement (see Fig. 1) can be performed. To elongate the femoral neck, the remaining base of the greater trochanter has to be reduced (see Fig. 1). To avoid damage to the nutrient vessel of the femoral head, the retinacular flap is developed. **a** Schematic drawing of developed flap and after offset correction. **b** Intraoperative image of retinacular flap and after offset correction. The flap includes the medial femoral circumflex artery, periosteum and the insertions of the short external rotators and ends at the head neck junction, where the retinacular vessels perforate the head. The reduction of the enlarged and aspherical femoral head with a head reduction osteotomy, also requires the development of the retinacular flap to secure head perfusion. Following correction of intra- and extraarticular impingement, range of motion is dynamically tested during surgical hip dislocation and additional correction is performed if needed. (From [17], with kind permission from SAGE Publishing)

**Fig. 9:**
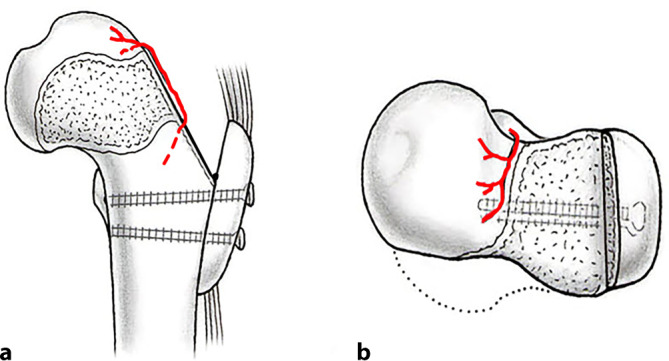
Closure of the capsule is followed by refixation of the trochanter in the distalized position with two 3.5 mm cortical screws, as can be seen in the a. p. view (**a**) and from above (**b**). Afterwards soft tissue and skin closure is performed

**Fig. 10:**
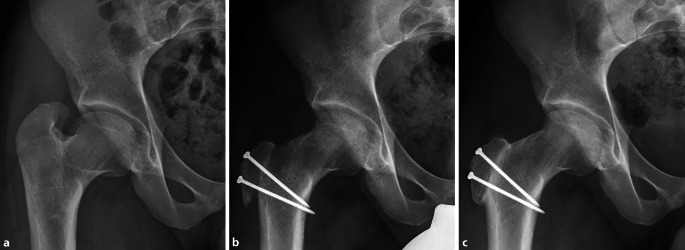
**a** Example of a 30-year-old man with typical femoral head deformity following LCPD (Legg-Calve-Perthes Disease) with an enlarged, aspherical head and a shortened neck. This results in a relatively high riding greater and lesser trochanter resulting in restricted range of motion and extraarticular impingement. **b** Following relative femoral neck lengthening, the greater trochanter was distalized, the base of it reduced to elongate the neck, the cam deformity was resected and in addition the lesser trochanter was distalized. Radiograph was performed at the 8‑week follow-up. **c** Radiograph at 1‑year following surgery with healed osteotomies, no signs of osteoarthritis or avascular necrosis

Figs. 7, 8, 9, and 10.

## Special surgical considerations

Surgical hip dislocation gives an excellent view of the joint to inspect and treat the femoral head, the head–neck junction, acetabulum, damaged cartilage or labrum, and concomitant pathologies to improve joint containment. During surgery, the impingement-free range of motion can be directly evaluated. The retinacular flap allows for correction of the neck while protecting femoral head perfusion. Precise knowledge of the vascular anatomy of the proximal femur is of upmost importance to perform this technically demanding surgery.

## Postoperative management

The postoperative protocol includes the use of a continuous passive motion device during the hospital stay, beginning directly postoperatively, to prevent capsular adhesions. After surgery, patients are mobilized with partial weight bearing of 15 kg with the use of crutches for at least 8 weeks. Depending on the performed procedures (head reduction, femoral or acetabular osteotomies) other restrictions may apply. Abduction, adduction as well as flexion more than 90° is restricted to protect the trochanteric osteotomy. Prophylaxis against thrombosis is prescribed until full weight bearing is allowed. Prophylaxis for heterotopic ossification is not applied on a regular basis, as this complication does not occur very often. After radiographic confirmation of healing at the 8‑week follow-up, stepwise return to full weight bearing is allowed and abductor training is initiated. Usually, return to work is possible 3 months postoperative.

## Errors, hazards, complications

- Iatrogenic lesion to the retinacular vessels with damage to vascular perfusion of the femoral head and avascular necrosis of the femoral head
- Intraarticular adhesions (hip arthroscopy might be necessary)
- Delayed union or pseudarthrosis of the trochanteric osteotomy can necessitate revision surgery
- Hinged abduction due to enlarged and aspherical femoral head (additional head reduction osteotomy might be necessary)
- Failure to achieve stability and joint containment (additional periacetabular osteotomy might be necessary)
- Heterotopic ossifications
- Muscular imbalance, change of gait pattern requiring long rehabilitation

## Results

In a retrospective case series, we evaluated 81 adult patients with history of LCPD, who underwent surgical hip dislocation with relative femoral neck lengthening and retinacular soft-tissue flap (Table 1).

**Table 1:** Demographics of evaluated patients with surgical hip dislocation

Parameter	Number
*Total number of hips (patients)*	81 (79)
*Tönnis grade preoperative (hips)*	
Stage 0	41
Stage 1	40
*Age at operation (years)*	23 ± 7 (6–51)
*Gender (% male)*	53%
*Side (% left)*	56%

### Preoperative findings

All 81 hips presented with clinical relevant intra- and/or extraarticular impingement following LCPD. Reduced range of motion and pain were the leading symptoms. Of the 81 hips, 41 had preoperative Tönnis stage 0 and 40 hips had Tönnis stage 1. In addition, 46 of the hips had previous operations, mostly intertrochanteric varisation osteotomies.

### Procedures

Between October 1997 and October 2020, 81 hips (79 patients) were treated with surgical hip dislocation and offset correction. In 71 hips, a retinacular flap with relative femoral neck lengthening was performed. Concomitant procedures were performed in 32 hips for the labrum, in 11 hips with a periacetabular osteotomy, in 6 hips with a valgus osteotomy, and in 4 hips with a head reduction osteotomy. The mean age at operation was 23 ± 9 (6–51) years.

### Follow-up and evaluation

The mean follow-up was 9 ± 7 (range 1–23) years; 11 hips were converted to a total hip arthroplasty (THA) due to progressive arthrosis after a mean 7 ± 4 (range 1–13) years. One patient died 1 year after the operation unrelated to surgery. The remaining 67 hips showed no or minor progression of the arthrosis.

### Revision surgery and complications

None of the patients developed avascular necrosis of the femoral head. Complications included two instabilities with consecutive subluxations, and one patient with pseudarthrosis of the lesser trochanter. Of the 2 patients with instabilities, one received a periacetabular osteotomy and the other patient underwent total hip arthroplasty due to advanced osteoarthritis. The patient with the pseudarthrosis of the lesser trochanter had a revision osteosynthesis and healed trochanter at follow-up. In 43 patients, the trochanteric screws had to be removed. One patient died unrelated to the operation.

### Conclusions

Our findings confirm earlier studies that surgical hip dislocation with relative femoral neck lengthening and retinacular soft-tissue flap is an effective treatment option for patients with post-Legg–Calve–Perthes (LCP) deformities [1]. The frequency of pseudarthrosis of the greater or lesser trochanter was similar to the frequency in patients with surgical hip dislocation without relative femoral neck lengthening [2]. The percentage of hips that were converted to THA (14%) is slightly higher than in other studies on results after relative femoral neck lengthening (7% in a follow-up of 3 years [2] and 10% in a follow-up of 3.75 years [15]) but seems to match the results of the other studies regarding the longer follow-up time of 9 years.

#### Infobox explanation of scores

*Waldenström:* The classification according to Waldenström groups the disease by typical radiographic signs into five major stages with substages:

- 1) Initial stage with augmentation of the joint space and joint effusion,
- 2) Condensation stage with sclerosis of the epiphysis (3–6 months after disease onset),
- 3) Fragmentation stage with depletion of the bone with cloddy decay (maximum 12 months after onset),
- 4) Regeneration stage with rebuilding of the bony substance (1–3 years after onset) and
- 5) End stage with completion of the bony healing with restitutio ad integrum or deformed healing.

*Tönnis:* Radiographic classification of osteoarthritis.

- Grade 0: No arthritis,
- Grade 1: Mild thinning of joint space and increased density of supporting bone,
- Grade 2: More thinning of joint space, small cysts, more density of supporting bone,
- Grade 3: Large cysts, severe narrowing or obliteration of joint space, deformity of femoral head.

## Declarations

Ethical review board approval was obtained for this study. All patients provided written consent and agreed to attend regular follow-up examinations.
